# Supplementary material for: Identification of photoperiod-induced specific miRNAs in the adrenal glands of Sunite sheep (Ovis aries)
Source: Front Vet Sci. 2022 Jul 22;9:888207. doi: 10.3389/fvets.2022.888207 (PMC9354845; doi:10.3389/fvets.2022.888207)
Supplement: Supplementary file 1 [file Data_Sheet_1.ZIP › Supplementary file/miRDeep2 file.zip!/miRDeep2 file/Know_Structure/SPLP42A3/oar-mir-125b.pdf]

miRBase precursor : oar-mir-125b
Total read count : 75795
oar-miR-125b read count : 74880
remaining reads : 915

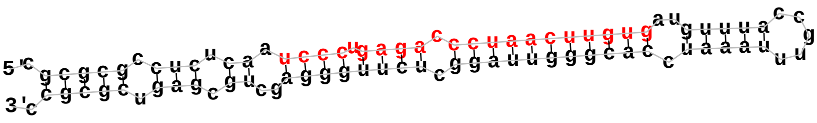

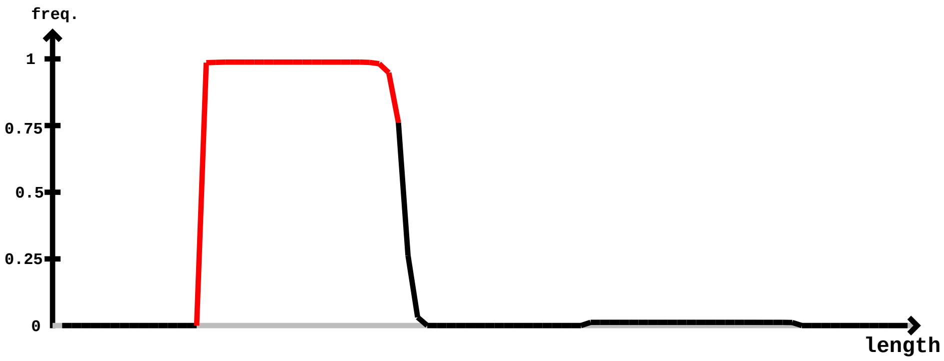


```
                oar-miR-125b
5'- cgcgcgccucucaaucccugagacccuaacuugugauguuuaccguuuaaauccacggguuaggcucuugggagcugcgagucgcgcc   -3'    exp
    .(((((.(((.((.((((.((((.((((((((((..(((((.....))))).))))))))))).))))))))..)).))).))))).    reads    mm    sample
    .............aucccugagacccuaacuu.........................................................  1        0     seq
    .............aucccugagacccuaacuugug......................................................  2        0     seq
    ..............ucccugagacccuaacu..........................................................  77       0     seq
    ..............ucccugagacccuaacC..........................................................  12       1     seq
    ..............ucccugagUcccuaacu..........................................................  1        1     seq
    ..............ucccugagacccuaacCu.........................................................  2        1     seq
    ..............ucccugagacccuaacuu.........................................................  365      0     seq
    ..............ucccugagacccuaacuug........................................................  2555     0     seq
    ..............ucccugagacccuaacuugu.......................................................  14154    0     seq
    ..............ucccugagacccuaacuugug......................................................  37743    0     seq
    ..............ucccugagacccuaacuuguga.....................................................  17461    0     seq
    ..............ucccugagacccuaacuugugaG....................................................  638      1     seq
    ..............ucccugagacccuaacuugugau....................................................  1696     0     seq
    ..............ucccugagacccuaacuugugaGg...................................................  3        1     seq
    ..............ucccugagacccuaacuugugaug...................................................  7        0     seq
    ..............ucccugagacccuaacuugugaugu..................................................  2        0     seq
    ...............cccugagacccuaacuug........................................................  1        0     seq
    ...............cccugagacccuaacuugu.......................................................  7        0     seq
    ...............cccugagacccuaacuugug......................................................  8        0     seq
    ...............cccugagacccuaacuuguga.....................................................  57       0     seq
    ...............cccugagacccuaacuugugau....................................................  4        0     seq
    ...............cccugagacccuaacuugugaG....................................................  3        1     seq
    ................ccugagacccuaacuug........................................................  2        0     seq
    ................ccugagacccuaacuugu.......................................................  18       0     seq
    ................ccugagacccuaacuugug......................................................  36       0     seq
    ................ccugagacccuaacuuguga.....................................................  20       0     seq
    ................ccugagacccuaacuugugaG....................................................  1        1     seq
    .................cugagacccuaacuugug......................................................  1        0     seq
    .................cugagacccuaacuuguga.....................................................  1        0     seq
    ..................ugagacccuaacuugug......................................................  1        0     seq
    ..................ugagacccuaacuuguga.....................................................  1        0     seq
    ......................................................cacggguuaggcucuugggagcu............  1        0     seq
    .......................................................acggguuaggcucuuggg................  3        0     seq
    .......................................................acggguuaggcucuugggagc.............  48       0     seq
    .......................................................acggguuaggcucuugggagcu............  859      0     seq
```

oar-miR-125b

```
cgcgcgccucucaaucccugagacccuaacuugugauguuuaccguuuaaauccacggguuaggcucuugggagcugcgagucgcgcc
......................................................acggguuaggcucuugggagcug...........    1          0          seq
.......................................................cggguuaggcucuugggagcu............    3          0          seq
```
